# An e-Learning Program for Physiotherapists to Manage Knee Osteoarthritis Via Telehealth During the COVID-19 Pandemic: Real-World Evaluation Study Using Registration and Survey Data

**DOI:** 10.2196/30378

**Published:** 2021-12-01

**Authors:** Ana Elisa Serafim Jorge, Kim Louise Bennell, Alexander Jared Kimp, Penny Kate Campbell, Rana Shane Hinman

**Affiliations:** 1 Center for Biological and Health Sciences Department of Physiotherapy Federal University of São Carlos São Paulo Brazil; 2 Centre for Health, Exercise and Sports Medicine Department of Physiotherapy, School of Health Sciences The University of Melbourne Melbourne Australia

**Keywords:** osteoarthritis, knee, physiotherapy, exercise, e-learning, telehealth, pain, education, implementation, evaluation, professional development, rehabilitation

## Abstract

**Background:**

The COVID-19 pandemic necessitated clinicians to transition to telehealth, often with little preparation or training. The Physiotherapy Exercise and Physical Activity for Knee Osteoarthritis (PEAK) e-learning modules were developed to upskill physiotherapists in management of knee osteoarthritis (OA) via telehealth and in-person. In the research setting, the e-learning modules are perceived by physiotherapists as effective when they are part of a comprehensive training program for a clinical trial. However, the effectiveness of the modules on their own in a *real-world* setting is unknown.

**Objective:**

This study aims to evaluate the reach, effectiveness, adoption, and implementation of PEAK e-learning modules.

**Methods:**

This longitudinal study was informed by the Reach, Effectiveness, Adoption, Implementation, and Maintenance (RE-AIM) framework. Participants were clinicians, researchers, educators, and health care students who registered for access to the modules between April 1 and November 30, 2020. Reach was evaluated by outcomes (countries, referral sources, and attrition) extracted from registration data and embedded within precourse surveys in the Learning Management System (LMS). Effectiveness was evaluated by outcomes (confidence with videoconferencing; likelihood of using education, strengthening exercise, and physical activity in a treatment plan for knee OA; usefulness of modules) measured using a 10-point numeric rating scale (NRS; score range from 1=not confident or likely or useful at all to 10=extremely confident or likely or useful) in pre- and postcourse (on completion) surveys in the LMS. Adoption and implementation were evaluated by demographic and professional characteristics and outcomes related to the use of learning and usefulness of program elements (measured via a 4-point Likert scale, from not at all useful to extremely useful) in a survey administered 4 months after module completion.

**Results:**

Broad reach was achieved, with 6720 people from 97 countries registering for access. Among registrants, there were high levels of attrition, with 36.65% (2463/6720) commencing the program and precourse survey and 19.61% (1318/6720) completing all modules and the postcourse survey. The program was effective. Learners who completed the modules demonstrated increased confidence with videoconferencing (mean change 3.1, 95% CI 3.0-3.3 NRS units) and increased likelihood of using education, strengthening and physical activity in a knee OA treatment plan, compared to precourse. Adoption and implementation of learning (n=149 respondents) occurred at 4 months. More than half of the respondents used their learning to structure in-person consultations with patients (80/142, 56.3%) and patient information booklets in their clinical practice (75/142, 52.8%).

**Conclusions:**

Findings provide evidence of the reach and effectiveness of an asynchronous self-directed e-learning program in a real-world setting among physiotherapists. The e-learning modules offer clinicians an accessible educational course to learn about best-practice knee OA management, including telehealth delivery via videoconferencing. Attrition across the e-learning program highlights the challenges of keeping learners engaged in self-directed web-based learning.

## Introduction

### Background

The prevalence of knee osteoarthritis (OA) is rapidly increasing as the population ages and the obesity epidemic rises worldwide [1]. As a debilitating chronic musculoskeletal condition, OA is ranked as the 12th leading cause of disability worldwide [2]. Education and exercise are highly recommended as core interventions [3-6], and these are often delivered via in-person consultations by physiotherapists [7,8]. In 2020, the COVID-19 pandemic imposed a substantial change in the delivery of in-person health care services worldwide [9,10]. Social distancing requirements and lockdowns necessitated many physiotherapists to rapidly transition to telehealth service delivery using freely available real-time videoconferencing software (eg, Zoom, Zoom Video Communications; Microsoft Teams, Microsoft; WhatsApp, Facebook Inc; and FaceTime, Apple Inc) [11,12], often with little preparation or training. A global survey of allied health practitioners between April and June 2020 showed that 68% of respondents used telehealth to manage people with OA during the pandemic and that exercise and education were used by 96% of respondents as part of telehealth consultations [11].

Telehealth is a term under the *digital practice* umbrella, which encompasses *health care services, support, and information* provided remotely via telecommunication technology [13]. Telehealth has been shown to be an effective model of physiotherapy service delivery for a range of musculoskeletal conditions [14], including OA [15]. A randomized controlled trial has shown the effectiveness [16] and patient acceptability [17] of the remote delivery of a physiotherapist-prescribed exercise program via video consultations for people with knee OA; however, before the COVID-19 pandemic, a minority of allied health clinicians provided telehealth services [11]. Before the pandemic, barriers to telehealth were multifactorial and included costs associated with implementing telehealth infrastructure, lack of third-party payer funding for telehealth consultations, and clinician resistance to practice change [15,18]. Significantly, many physiotherapists lack specific training in delivery of care via telehealth [13,19], and subsequently may have little knowledge [18], experience [20], and confidence [21] in telehealth delivery. Research shows that clinician education about and exposure to telehealth increases both acceptance of, and confidence with, delivery of care via telehealth [20,21]. Furthermore, physiotherapists and other health care professionals have highlighted limitations in their knowledge, skills, and confidence in managing people with knee OA [22]. Thus, providing education and knowledge resources to clinicians in telehealth skills to deliver evidence-based OA management is paramount.

e-learning is an accessible and scalable method for delivering health professional education and training. e-learning broadly relates to the delivery of educational material through information and communication technology, often using the internet to wholly or partially replace the need for a human instructor [23]. The Physiotherapy Exercise and Physical Activity for Knee Osteoarthritis (PEAK) e-learning modules [24] were developed by researchers, in the context of a clinical trial [25], to upskill physiotherapists in evidence-based management of knee OA through telehealth and in-person consultations. The self-directed modules aim to educate learners on how to implement evidence-based physiotherapy care using a structured program of education, strengthening exercises, and individualized physical activity over 5 individual consultations, delivered via videoconferencing or in-person. In the research setting, the PEAK e-learning modules were perceived by physiotherapists as effective when delivered as part of a comprehensive training program for a clinical trial [20]. However, the effectiveness of the PEAK e-learning modules on their own in a *real-world* setting is unknown.

### Objectives

In light of the unfolding COVID-19 pandemic, the PEAK e-learning modules were released globally, free of charge, on April 1, 2020, to assist physiotherapists and other clinicians in providing care to patients with knee OA through telehealth. There is currently limited research evaluating professional development initiatives (ie, beyond entry-to-practice training) for physiotherapists regarding the management of OA. Guided by the Reach, Effectiveness, Adoption, Implementation and Maintenance (RE-AIM) framework [26], this study aims to evaluate the reach, effectiveness, adoption, and implementation of the PEAK e-learning modules during the COVID-19 pandemic. Specifically, we aimed to (1) evaluate the reach of, and attrition across the PEAK e-learning program; (2) evaluate the effectiveness of the PEAK e-learning program in building confidence with telehealth and intention to use core recommended OA treatments among learners who completed the program; and (3) evaluate how learners who completed the PEAK e-learning program implemented what they learned into practice 4 months after completion, including the settings it was adopted into.

## Methods

### Design

A longitudinal study with pre- and postcourse evaluations was conducted via an electronic survey. The RE-AIM framework [26] has informed the study.

### Participants

Participants in this study were those who registered for access to the PEAK e-learning modules between April 1, 2020 (launch date), and November 30, 2020. There were no specific eligibility criteria for registration (learners self-select whether to register based on the English language description of the e-learning modules on the registration page) and no costs were charged for the modules; thus, participants in this study included health care clinicians, researchers, educators, and health care students from anywhere in the world. After activating access to the Learning Management System (LMS), learners provided consent for researchers to use their program data for research purposes. Four months after the completion of the e-learning modules, learners were sent an electronic survey about how they had implemented their learnings into practice. Completion of this survey was voluntary, and implied consent to participate. This study was approved by the University of Melbourne Human Research Ethics Committee (#2056938).

### The PEAK e-Learning Modules

The PEAK e-learning modules [24] teach clinicians how to deliver an evidence-based management program (PEAK program) for people with knee OA, either via telehealth (videoconferencing) or in-person. The PEAK program and the e-learning modules were devised by researchers at the University of Melbourne specifically for use in a National Health and Medical Research Council–funded clinical trial [25] comparing videoconferencing with face-to-face care by physiotherapists for people with knee OA (trial ongoing). Thus, the program was designed for delivery by physiotherapists in Australia but is relevant to physiotherapists and other health care clinicians globally. The program focuses on education, strengthening exercise, and physical activity, delivered during 5 consultations over 3 months and can be individualized to patient needs. The e-learning modules were made publicly available across the globe on April 1, 2020, and they were promoted by researchers via social media and directly to physiotherapy professional organizations worldwide.

The asynchronous e-learning modules were delivered on the University of Melbourne LMS (Canvas LMS by Instructure, 2019), covering (1) evidence-based best-practice knee OA management, (2) telehealth (the delivery of care via Zoom videoconferencing), and (3) the PEAK program (a structured physiotherapy treatment protocol). Each module included a quiz at the end to help reinforce learning. The modules were sequentially released in the order listed, with access to subsequent modules unlocked as the preceding module was completed (defined as all pages viewed within the module and quiz questions submitted). Quizzes required >80% correct answers for advancement to the next module, and learners were allowed unlimited, multiple attempts at quiz questions. Learners were instructed to allow approximately 3 to 4 hours to complete all modules. The e-learning modules also provided learners with access to a website of videos of the exercises contained within the PEAK program. For learners who completed all e-learning modules, a suite of resources was unlocked on completion for printing and downloading. This included educational booklets that clinicians can provide to their patients (*Preparing for your Consultations*, *Osteoarthritis Information*, *Exercise Booklet*, and *Knee Plan and Log Book*) as well as clinician resources (*Zoom Troubleshoot Guide*, *Initiating and using Zoom for video consultations, Accessing the website of exercise videos, Pre-consultation survey, Consultation Outline, and Readiness Checklist*). On completion of the e-learning modules, learners could request a certificate of completion via email.

Users registered for access via a web-based form (Qualtrics International) housed at the University website [24], where they provided their names and email addresses. It took up to 24 to 48 hours after registration for the research staff to create a log-in. External users (non–University staff or students) were required to activate their account before they could log into the LMS to commence learning.

### Data Collection

#### Registration Data

The number of registrants was captured using the Qualtrics registration form. The geographic location of registrants was estimated from the approximate longitude and latitude obtained from their deidentified IP addresses. Researchers used the location data to correlate this with known country and continent information.

#### LMS Data

The PEAK e-learning modules contained pre- and postcourse survey questions (Multimedia Appendix 1) embedded within the LMS. After activating their LMS account, learners were provided with an introduction to the e-learning modules and completed a mandatory precourse survey before the first module was accessible. Learners who completed all the modules were invited to complete a postcourse survey. As an incentive, the printable and downloadable resources and instructions for obtaining a certificate of completion were made available to those who completed the postcourse survey. No fee was charged to obtain a certificate of completion.

The precourse survey comprised brief descriptive questions regarding learners’ professional characteristics and their usual clinical practice. Precourse levels of *confidence* with videoconferencing and *likelihood to use* education and strengthening exercise and physical activity in a treatment plan for patients with knee OA were ascertained by a series of questions each rated via a 10-point numeric rating scale (NRS), ranging from 1=not at all confident or likely to 10=extremely confident or likely. The postcourse survey immediately reassessed these questions, with additional questions about *how long it took to complete the modules* (via dropdown lists for hours and minutes) and *how useful was the course* (NRS 1=not useful at all to 10=extremely useful).

#### Adoption and Implementation Data

Learners who completed the postcourse survey within the LMS were invited to complete another survey 4 months later to ascertain the adoption and implementation of learnings into practice. This electronic survey (Multimedia Appendix 2) link was sent by email and accessed via a secure web-based survey tool (REDCap, Research Electronic Data Capture, Vanderbilt University). The survey presented slightly different questions depending on whether the learner was a health professional, student, an educator, or a researcher. The first section ascertained demographic information and professional characteristics (data regarding adoption). The second section ascertained what the learner had implemented into practice from the PEAK e-learning modules, including how they used the downloadable resources. Questions evaluated the usefulness of the PEAK e-learning modules and their resources, as well as the extent to which learnings from the modules had changed learners’ practice and how. The barriers to implementation were also evaluated. Learners rated their agreement with relevant statements using a 4-point Likert scale ranging from *not at all* to *extremely useful* or *to a large extent*.

### Data Analysis

Data were downloaded from the LMS and REDCap software and processed in Excel (Microsoft Corporation). Descriptive analysis of the data was performed using means, SD, and proportions with Excel, where appropriate. For the second aim, only data from individuals who had answered both the precourse and postcourse surveys were analyzed for paired outcomes. For paired outcomes, individual change scores (for each learner and each outcome) were calculated by subtracting precourse scores from postcourse scores. The mean change (95% CI) was then calculated for each outcome.

## Results

### Aim 1: Reach of, and Attrition Across, the PEAK e-Learning Modules

From launch (April 1) to November 30, 2020, 6720 people registered for access to the PEAK e-learning modules. There was a broad international reach. Registrants came from 97 countries, with the top 10 most common countries being Australia (2077/6720, 30.91%), Canada (870/6720, 12.95%), United Kingdom (636/6720, 9.46%), United States (632/6720, 9.40%), South Africa (253/6720, 3.76%), Ireland (251/6720, 3.74%), Romania (152/6720, 2.26%), India (148/6720, 2.20%), Brazil (121/6720, 1.80%), and New Zealand (119/6720, 1.77%). The 5 most common nationalities have English as their native language.

**Figure 1:**
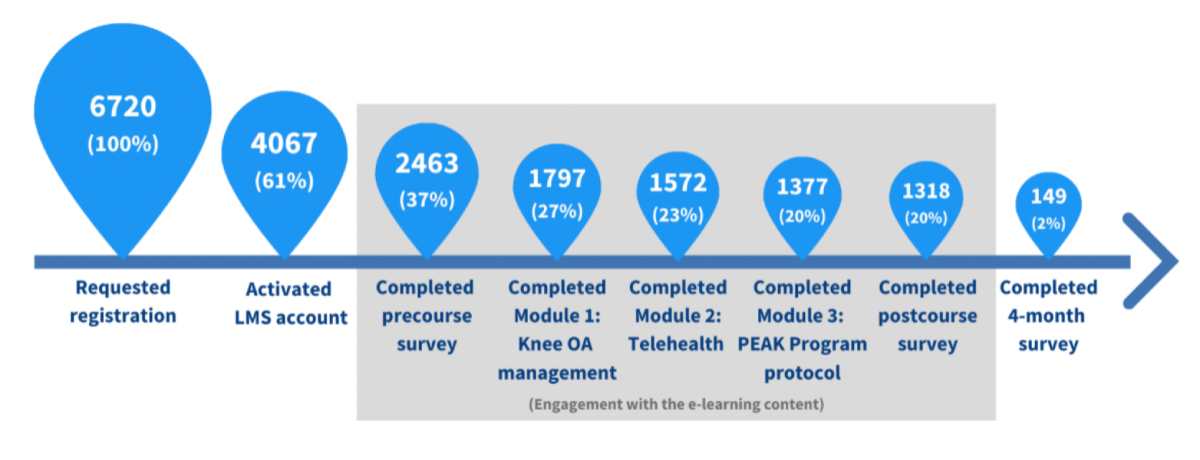
Users since program launch. Pipeline depicting the number of people who registered for access, activated their Learning Management System (LMS) account, and completed the various modules of the e-learning program. Percentages are calculated as a proportion of those who requested registration. OA: osteoarthritis.

There was attrition of learners across the pipeline from registration to the 4-month survey completion (Figure 1). The greatest attrition occurred between requesting registration and activating the LMS account, with 39.48% (2653/6720) of registrants failing to activate their LMS account. Of the 4067 users who activated their account within the LMS, 60.56% (2463/4067) completed the precourse survey, 44.18% (1797/4067) completed the first module, and 32.41% (1318/4067) completed all modules as well as the postcourse survey. Of the 2463 learners who completed the precourse survey, 53.51% (1318/2463) completed all modules and the postcourse survey, but only 6.05% (149/2463) completed the 4-month survey.

Of the participants who activated their LMS account and completed the precourse survey, most found out about the PEAK e-learning modules through a work colleague (795/2463, 32.28%). Other referral sources included Twitter (450/2463, 18.27%), professional organizations (368/2463, 14.94%), Facebook (281/2463, 11.41%), the internet (132/2463, 5.36%), a physiotherapy course instructor (128/2463, 5.20%), a friend/family (114/2463, 4.63%), other health professional course instructor (31/2463, 1.26%), LinkedIn (23/2463, 0.93%), and other sources (134/2463, 5.44%).

The characteristics of learners who completed the e-learning program (ie, those who completed both the pre- and postcourse surveys) and noncompleters (ie, those who completed the precourse survey but not the postcourse survey), along with those who completed the 4-month survey (n=149) are summarized in Tables 1 and 2. The characteristics were generally similar across completers and noncompleters, except that more Australians tended to complete the program, whereas other geographic locations had similar proportions of completers and noncompleters. Similarly, there was a higher proportion of completers than noncompleters among chiropractors compared with other professions. The small sample of respondents to the 4-month survey was largely physiotherapists, predominantly from Australia.

**Table 1:** Demographic characteristics of learners who completed (completers) and did not complete (noncompleters) the e-learning program.

Variables		Completers (n=1318)^a^	Noncompleters (n=1145)^a^	Completed 4-month survey (n=149)^a^
**Gender, n (%)**				
	Female	—^b^	—	93 (62)
	Male	—	—	54 (36)
	Do not wish to disclose	—	—	2 (1)
**Age (years), mean (SD)**				
	≤30	—	—	29 (19)
	51-60	—	—	41 (28)
	≥60	—	—	33 (22)
	31-40	—	—	16 (11)
	41-50	—	—	4 (3)
**Main work role, n (%)^c^**				
	Physiotherapist delivering clinical care to patients	869 (66)	796 (70)	121 (81)
	Physiotherapy student	159 (12)	100 (9)	4 (3)
	Other health professional student	118 (9)	43 (4)	3 (2)
	Other health professional delivering clinical care to patients	74 (6)	61 (5)	14 (9)
	Other	27 (2)	54 (5)	0 (0)
	Education of physiotherapy students	23 (2)	51 (4)	2 (1)
	Physiotherapy researcher	20 (2)	28 (2)	2 (1)
	Education of other health professional students	24 (2)	8 (1)	0 (0)
	Other health professional researcher	3 (0)	3 (0)	3 (2)
**Location, n (%)^d^**				
	Africa	33 (3)	45 (4)	7 (5)
	Asia	53 (4)	96 (8)	12 (8)
	Australia	549 (42)	373 (33)	61 (41)
	Europe	261 (20)	265 (23)	27 (18)
	North America	372 (28)	302 (26)	30 (20)
	Pacific islands	36 (3)	24 (2)	8 (5)
	South America	13 (1)	37 (3)	4 (3)

^a^Individual characteristics may not add to totals due to missing data.

^b^—: not recorded.

^c^Percentages calculated for completers and noncompleters based on n=1317 and 1144, respectively.

^d^Percentages calculated for completers and noncompleters based on n=1317 and 1142, respectively.

**Table 2:** Clinical practice characteristics of learners who completed (completers) and did not complete (noncompleters) the e-learning program.

			Completers (n=1318)^a^	Noncompleters (n=1145)^a^	Completed 4-month survey (n=149)^a^		
**Profession, n (%)^b^**							
		Physiotherapist/physical therapist	782 (78)	692 (84)	126 (89)		
		Chiropractor	147 (15)	54 (7)	5 (3)		
		Rheumatologist	3 (0)	1 (0)	0 (0)		
		General practitioner or family physician	0 (0)	2 (0)	0 (0)		
		Sport and exercise medicine physician	0 (0)	3 (0)	0 (0)		
		Orthopedic surgeon	0 (0)	2 (0)	1 (1)		
		Dietitian	1 (0)	0 (0)	0 (0)		
		Podiatrist	1 (0)	0 (0)	0 (0)		
		Other	67 (7)	70 (8)	10 (7)		
Average patients with knee OA^c^ treated per month, mean (SD)			12 (17)	12 (38)	8 (5)		
Clinical practice experience (years), mean (SD)			9.4 (10.2)	10.0 (9.9)	—^d^		
**Strategies usually used to manage people with knee OA, n (%)**							
	Education		1218 (92)	1051 (92)	—		
	Exercise		1242 (94)	1092 (95)	—		
	Physical activity advice		1164 (88)	1003 (88)	—		
	Weight loss advice		887 (67)	746 (65)	—		
	Manual therapy		610 (46)	550 (48)	—		
	Acupuncture		110 (8)	91 (8)	—		
	Bracing		147 (11)	145 (13)	—		
	Shoe orthotics		130 (10)	134 (12)	—		
	Other		174 (13)	137 (12)	—		
**Currently offer videoconferencing consultations to patients with knee OA, n (%)^e^**							
	Yes		366 (28)	317 (28)	72 (51)		
	No		939 (72)	817 (72)	68 (49)		
Experience with teleconsultations using videoconferencing^f^, mean (SD)			2.4 (1.9)	2.3 (1.9)	—		

^a^Individual characteristics may not add to totals due to missing data.

^b^Percentages calculated for completers, noncompleters, and 4-month completion based on n=1001, 824, and 142, respectively.

^c^OA: osteoarthritis.

^d^—: not recorded.

^e^Percentages calculated for completers, noncompleters, and 4-month completion based on n=1305, 1134, and 140, respectively.

^f^Scored on a 10-point numerical rating scale (1=no experience at all to 10=extremely experienced).

### Aim 2: Effectiveness of the PEAK e-Learning Modules

**Figure 2:**
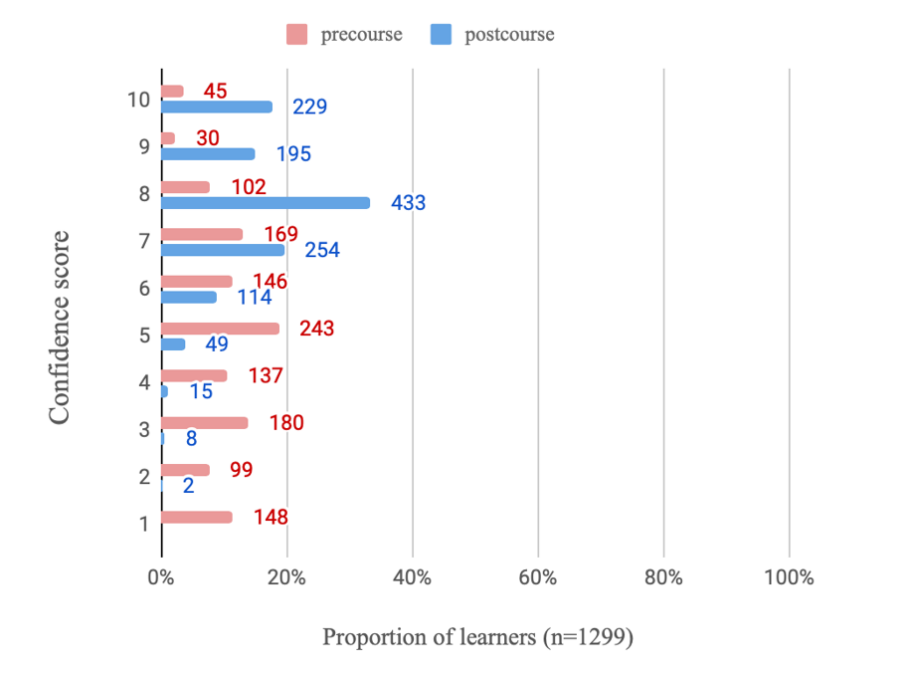
Distribution of confidence scores with videoconferencing consultations for participants (n=1299) who answered this question pre- and postcourse (where scores of 1=not confident at all and 10=extremely confident).

**Figure 3:**
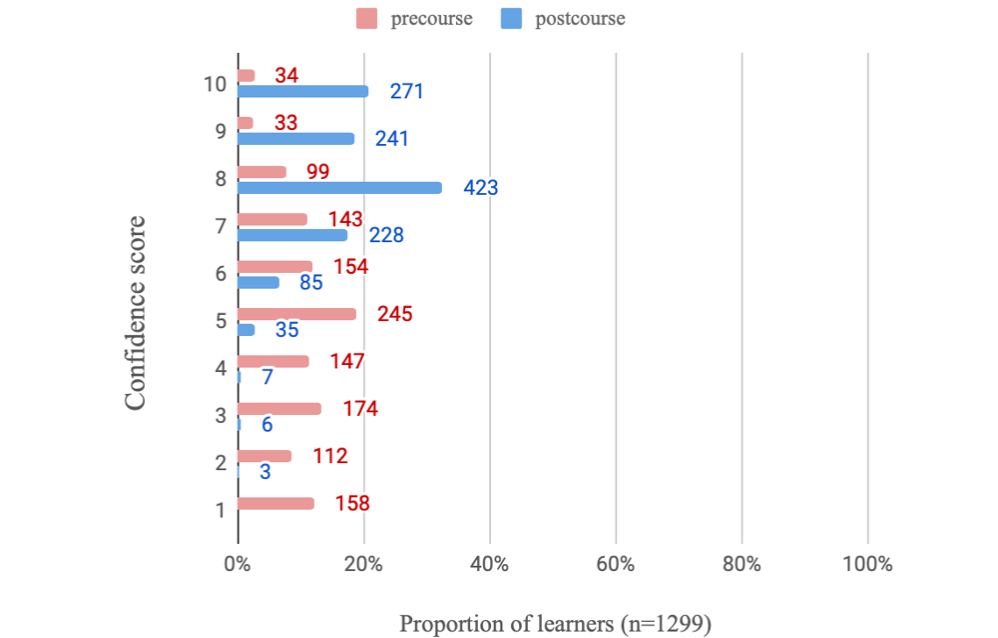
Distribution of confidence scores with videoconferencing consultations specifically for management of knee osteoarthritis for participants (n=1299) who answered this question pre- and postcourse (where scores of 1=not confident at all and 10=extremely confident).

**Figure 4:**
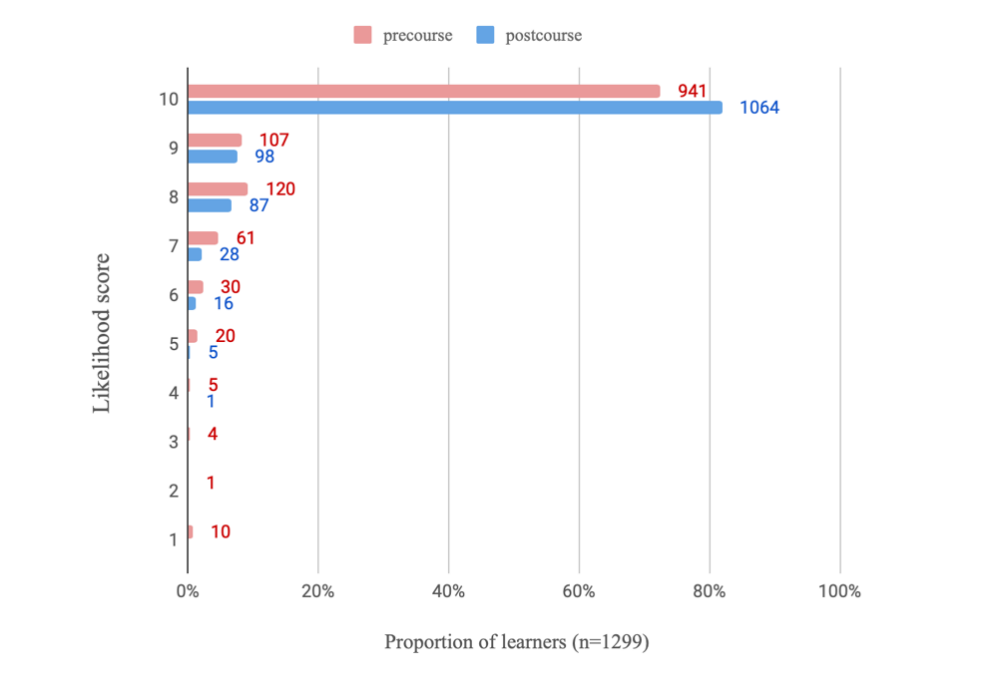
Distribution of scores (n=1299) regarding likelihood to use education in a treatment plan for patients with knee osteoarthritis (where scores of 1=not at all likely and 10=extremely likely).

**Figure 5:**
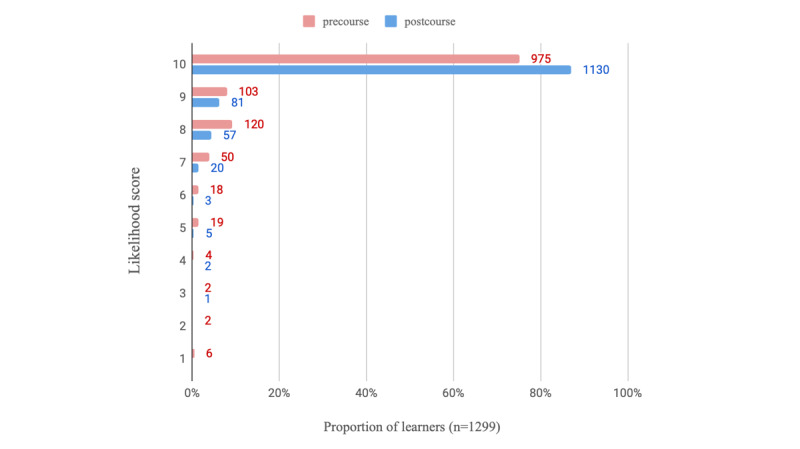
Distribution of scores (n=1299) regarding likelihood to use strengthening exercise in a treatment plan for patients with knee osteoarthritis (where scores of 1=not at all likely and 10=extremely likely).

**Figure 6:**
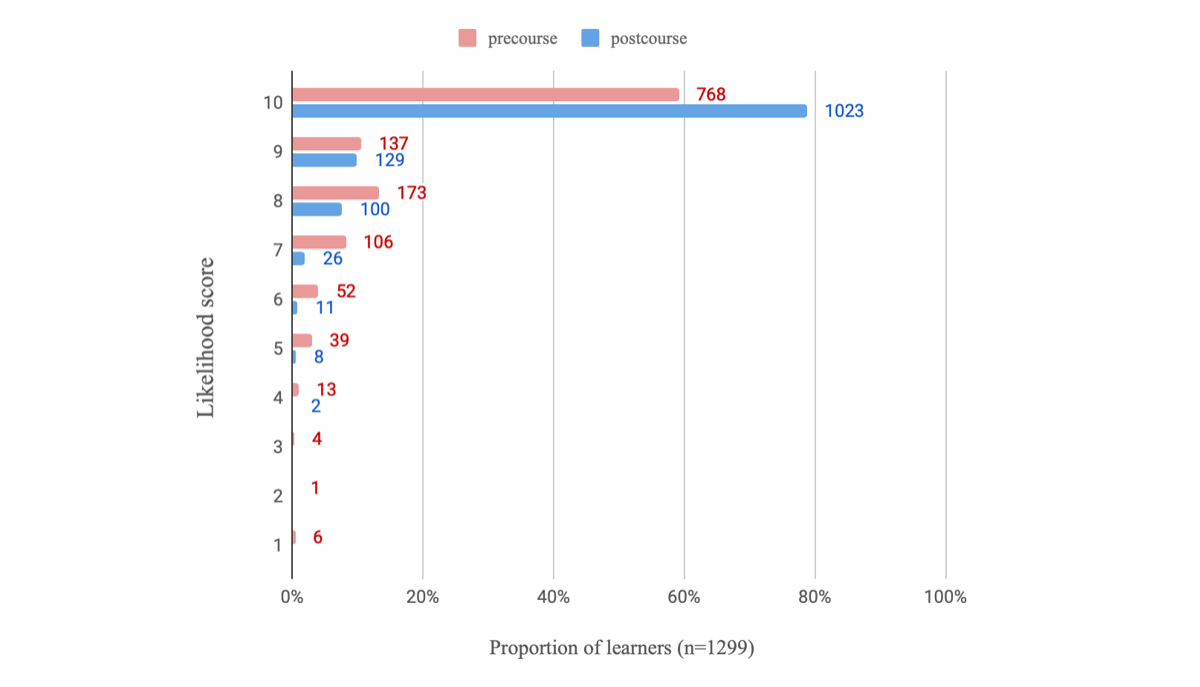
Distribution of scores (n=1299) regarding likelihood to use physical activity in a treatment plan for patients with knee osteoarthritis (where scores of 1=not at all likely and 10=extremely likely).

On average, learners who completed all modules and the postcourse survey spent a mean of 4.1 (SD 2.7) hours using the e-learning program (n=1318 respondents). Regarding the usefulness of the PEAK e-learning modules (n=1317 respondents), learners scored on average, 8.7 (SD 1.4) on a 10-point NRS, indicating high levels of perceived usefulness. The learners who completed the course reported increased confidence in videoconferencing (Table 3) relative to precourse. This is visualized in Figure 2 and Figure 3, where regarding confidence in using videoconferencing consultations with patients with knee OA, 71.98% (935/1299) of completers scored at least 8 out of 10 on the NRS postcourse compared with just 13.00% (166/1277) before. The likelihood of using education, strengthening exercise, and physical activity in a treatment plan for people with knee OA also increased postcourse (Table 3). Figures 4-6 graphically display the distribution of scores, with the greatest shifts in NRS scores postcourse occurring with the likelihood of using physical activity in a treatment plan. Precourse, 82.99% (1078/1299) of learners were likely to use physical activity in a treatment plan, compared with 96.01% (1252/1304) postcourse.

**Table 3:** Immediate changes in confidence with videoconferencing and likelihood to use education, strengthening exercise, and physical activity in a treatment plan for patients with knee osteoarthritis in learners (n=1299) who answered both the pre- and postcourse surveys.

	Precourse^a^, mean (SD)	Postcourse^a^, mean (SD)	Mean change (95% CI)^b^
Confidence with videoconferencing	4.8 (2.4)	7.9 (1.5)	3.1 (3.0-3.3)
Confidence with videoconferencing for people with knee OA^c^	4.7 (2.4)	8.2 (1.4)	3.5 (3.4-3.6)
Likelihood to use education	9.3 (1.5)	9.7 (0.9)	0.4 (0.3-0.5)
Likelihood to use strengthening exercise	9.4 (1.3)	9.8 (0.7)	0.4 (0.3-0.5)
Likelihood to use physical activity	8.9 (1.6)	9.6 (0.9)	0.7 (0.6-0.8)

^a^Scored on a 10-point numeric rating scale (1=not at all confident/likely to 10=extremely confident/likely).

^b^Calculated as postcourse score minus precourse score.

^c^OA: osteoarthritis.

### Aim 3: Adoption and Implementation of Learnings From the PEAK e-Learning Modules at 4 Months

Demographic and clinical characteristics of the few learners who completed the 4-month adoption and implementation survey (n=149) are shown in Tables 1 and 2. Regarding health care setting, 48.3% (72/149) respondents were from private practice, 21.5% (32/149) from acute care hospitals, 10.7% (16/149) from rehabilitation hospitals, 16.1% (24/149) from community health center/settings, 1.3% (2/149) from Veterans Affairs settings, and 12.1% (18/149) from other settings. Musculoskeletal health care was the predominant area of clinical practice for respondents (106/149, 71.1%), followed by gerontology with 13.4% (20/149) of respondents.

**Figure 7:**
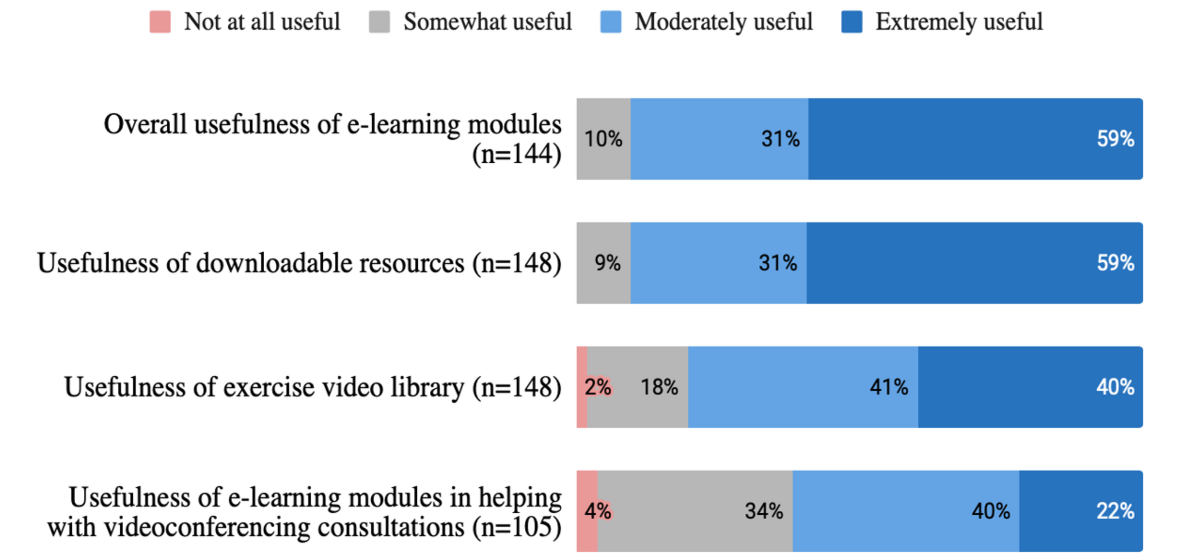
Perceptions of 4-month survey respondents about usefulness of learnings.

Figure 7 shows learner perceptions about the usefulness of the e-learning modules 4 months after completion of the course. More than half described the e-learning program (85/144, 59.0%) and its downloadable resources (88/149, 59.1%) as extremely useful. About 90.1% (128/142) of learners had recommended the PEAK e-learning modules and/or downloadable resources to others.

**Figure 8:**
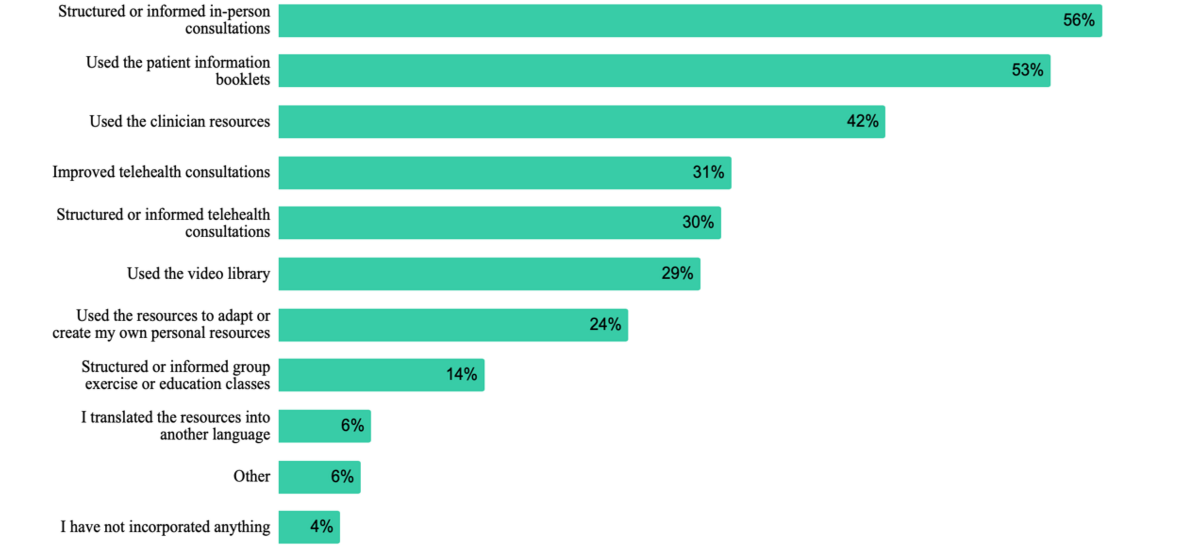
Strategies used by respondents (n=142) at 4 months to incorporate learnings into usual practice.

Almost all learners indicated that the e-learning modules had changed/informed their usual practice in some way, with 29.9% (44/147) of learners indicating their usual practice was changed to a minor extent, 55.1% (81/147) to a moderate extent, and 14.3% (21/147) to a large extent. Learnings from the e-learning modules were incorporated into clinical practice in a wide variety of ways (Figure 8). More than half of all respondents used their learnings to structure/inform in-person consultations with patients (80/142, 56.3%) and used the patient information booklets in their practice (75/142, 52.8%). Five learners had translated the resources into other languages (Chinese/Mandarin, Greek, Hungarian, Portuguese, and Spanish). Only 4.2% (6/142) of respondents indicated that they had not incorporated any learnings into their usual practice.

## Discussion

### Principal Findings

This study evaluated the reach, effectiveness, adoption, and implementation of learnings from the PEAK e-learning modules in a *real-world* context when the e-learning program is provided free of charge to users globally. Our findings showed a broad reach of the program from clinicians (mainly physiotherapists) across the world, predominantly driven through word of mouth, professional organizations, and social media. However, attrition was considerable from registration to the completion of the program. The learners who completed the course reported greater confidence in videoconferencing and increased likelihood of using education, strengthening exercises, and physical activity to manage knee OA. Four months after completion, learnings had been adopted in a range of musculoskeletal health care settings, predominantly by physiotherapists. Most respondents (85/144, 59.0%) to the follow-up survey described the e-learning program as extremely useful, and almost all (146/147, 99.3%) indicated that learnings had changed or informed their usual practice in some way. Collectively, these findings suggest that a self-directed e-learning approach may be an effective and scalable method for educating clinicians in a real-world context when resourcing for human instructors is not possible. Asynchronous e-learning programs also have additional advantages, including flexibility in pacing learning and overcoming the time and costs associated with travel by learners to attend educational courses [27].

The findings of this real-world evaluation are aligned with those of our previous qualitative evaluation of the training program in a research context [20]. The PEAK e-learning modules were originally developed by our team at the University of Melbourne to train 15 physiotherapists to deliver care in the context of a clinical trial [25] comparing videoconferencing with face-to-face care by physiotherapists for people with knee OA (ongoing). Training for the trial included not only the e-learning modules (as evaluated in this study) but also *practical* synchronous components, whereby physiotherapists participated in a mock initial consultation via videoconferencing with a physiotherapist researcher (AJK) followed by 4 practice video consultations with 2 pilot patients with chronic (>3 months) knee pain (recruited by research staff). Although there was 100% completion of the e-learning modules by the 15 physiotherapists in the PEAK trial, module completion was mandatory to deliver care in the trial, and physiotherapists were financially compensated for their time spent in training. Our qualitative study [20] exploring the trial physiotherapists’ experiences with the training program showed that physiotherapists valued the self-directed and self-paced nature of e-learning, even though it was unfamiliar to them. Similar to this study, trial physiotherapists reported increased confidence and ability to deliver care through telehealth. They valued the combined package of e-learning modules and the practical components. In contrast, this real-world evaluation focused only on the asynchronous e-learning modules. Despite the lack of structured practical learning components, learners who completed the modules reported a 74% increase in confidence with videoconferencing for people with knee OA, showing that the modules can be effective at scale without practical components of training. These findings are consistent with those of other research showing preliminary evidence of the effectiveness, acceptability, and feasibility of an e-learning program to educate physiotherapists to deliver a group-based self-management complex intervention for low back pain and OA [28].

There are 37,113 registered physiotherapists in Australia [29]. Thus, our 2077 registrants from Australia represent 6% of registered physiotherapists, suggesting a broad reach to this population. Several factors likely contributed to the broad reach of the PEAK e-learning modules. The COVID-19 pandemic has accelerated a rapid shift to telehealth service delivery for physiotherapy services [30,31]. We released the e-learning modules on April 1, 2020, as part of global efforts to facilitate adoption and implementation of physiotherapy via telehealth [10]. The content of the program is relevant to a wide number of clinical professions, as evident by learner enrollment data, particularly for professions that use exercise and physical activity to manage chronic diseases. Moreover, the telehealth module is relevant to any health professional. Although we did not collect data on why learners registered for the PEAK e-learning modules, it is highly likely that its focus on telehealth was of interest to many, particularly given that allied health clinicians worldwide have described inadequate training, resources, and confidence in telehealth delivery as a barrier to implementing telehealth services during the pandemic [11,32]. Educational courses are considered by many clinicians as a practice-changing phenomenon [27]. Our e-learning modules were free of charge to the learner, which probably also contributed to the large number of people registering for the learning program, spanning 97 countries. Financial constraints are known to be a barrier to participation in professional development among health professionals [33], particularly among those from resource-limited countries [34,35]. A recent systematic review showed that physiotherapists value time as well as accessible and trustworthy resources when undertaking learning and professional development activities [36]. Our e-learning modules, developed by expert researchers at a respected university, delivered at no cost over the internet and able to be undertaken in a self-paced manner aligns with these values. Indeed, a recent survey of 464 health care workers from Sub-Saharan Africa showed that web-based professional development opportunities were accepted and that self-paced internet or computer-based learning is a preferred learning modality [35].

Consistent with the literature showing greater dropout rates with web-based learning compared with traditional classes [37], we observed high levels of attrition across our e-learning program. The greatest attrition occurred between registration and account activation. Although 6720 people registered for access, only 60.52% (4067/6720) activated their account within the LMS. The reasons for attrition at this point are unclear but may be related to the unwieldy activation (several technical steps are required to create an account as an external user and potential security features such as firewalls/spam that blocked emails from the LMS platform) and navigation processes of the LMS, which were highlighted as barriers in our qualitative evaluation [20]. These factors are consistent with a systematic review of enablers and barriers affecting health sciences e-learning, in which one of the major barriers to e-learning is the lack of user-friendly technology [38]. Of the 4067 users who activated their account within the LMS, 61.00% (2481/4067) completed the precourse survey, 43.99% (1789/4067) completed the first module, 31.99% (1301/4067) completed all modules and the postcourse survey, showing relatively little attrition once learners engaged with the first module. The requirement to complete the precourse survey before commencing the first module may have been a barrier to progressing through the course. Only 9.00% (366/4067) of the learners with activated accounts dropped out from module 1 to module 2. Given that health professionals and students perceive that limitations in their knowledge and skills about OA are barriers to implementing OA care [22], and that clinicians have inadequate training, resources, and confidence in telehealth delivery [11,32], these factors may explain the high retention across the 3 modules. Overall, our findings appear consistent with the literature, where it is reported that 40% to 80% of students drop out of web-based classes [37]. Furthermore, physiotherapists express a preference for face-to-face workshops to address learning needs regarding the management of patients with persistent knee pain [39], regarding web-based learning formats as convenient, but not as effective as face-to-face learning. Given that the learners in our study came from 97 countries, many of which do not have English as the native language, it is likely that many experienced difficulties with the English language of the PEAK modules, probably contributing to the attrition we observed.

Our data show that the PEAK e-learning modules led to improved confidence in videoconferencing among learners, as well as an increased likelihood of using education, strengthening exercises, and physical activity to manage knee OA. The mean scores for the likelihood of using these interventions were already quite high precourse, with most learners (at least 88%) already using these strategies at the time they enrolled in the modules. This likely explains why the mean change in these scores was quite small. In contrast, confidence with videoconferencing was quite low among our sample precourse, and most (939/1305, 71.95%) were not offering videoconferencing to their patients with knee OA, leading to large improvements (64%-74%) in confidence outcomes postcourse. Interestingly, our adoption and implementation data (n=149) showed that 55.7% (83/149) of respondents used their learnings to structure or inform *in-person* consultations versus 30.2% (45/149) who used learnings to structure or inform *telehealth* consultations. It is not clear why there was a greater implementation of learnings in in-person consultations. However, this may be due to physiotherapists experiencing system-level and technological barriers to adopting and implementing telehealth during the pandemic [11,12,32] or related to the preferences of patients and health care professionals for in-person consultations [11,12].

From an implementation perspective, the proportion of respondents who reported using patient information booklets (79/149, 53.0%), clinician resources (63/149, 42.3%), and video library (43/149, 28.9%) from the PEAK program is noteworthy despite the small sample size (n=149). Furthermore, some of these resources had been translated into 5 languages by users. Twenty-four percent of respondents indicated that they created their own personal resources from the materials provided. These findings are relevant to other developers of e-learning programs for health professionals, highlighting the importance of embedding an *implementation tool kit*, which contains clinically relevant resources that can be used in patient interactions to facilitate care delivery.

### Strengths and Limitations

The strengths of our study include the evaluation of an e-learning program in a *real-world* context. This resulted in a broad reach across the globe, including low- and middle-income countries, as well as those where English is not the native language. Although the program was targeted to physiotherapists, our study participants included not only physiotherapists but also a range of health professionals as well as students, educators, and researchers. Limitations include our relatively short 4-month follow-up of adoption and implementation of learnings, and the low response rate (11% of those invited) to the 4-month email survey, leading to a small sample size (n=149). In addition, owing to the high attrition rate between pre- and postcourse surveys, our findings may overestimate the effectiveness of the PEAK e-learning modules, given that people who found the course useful may be more likely to have completed all modules and the postcourse survey than learners who did not find the modules useful. We also used custom-designed self-reported questions to determine changes in confidence and likelihood of using education, strengthening exercise, and physical activity in an OA treatment plan. As such, it is not clear if the improvements we observed pre- to postcourse are of *clinical* relevance. Future research should consider collecting patient-level data to determine whether improvements in clinician knowledge and confidence with e-learning translate into better clinical outcomes for patients with the health condition of interest. Although we did not develop the e-learning modules and embedded resources through formal co-design methods, we did refine the e-learning modules based on qualitative feedback from the physiotherapists who used the e-learning modules as mandatory training for the PEAK trial. The embedded patient and clinician resources were developed iteratively over many years by our research team, and consumers with OA provided feedback on resource content during this time. However, we may have seen less attrition with the e-learning modules had we co-designed these modules with physiotherapists at the outset.

### Conclusions

In conclusion, this study provides evidence of reach and effectiveness of an asynchronous e-learning program provided globally free of charge in a real-world setting among physiotherapists. The PEAK e-learning modules offer clinicians an accessible educational course to learn about best-practice knee OA management, including telehealth delivery via videoconferencing. Attrition across the e-learning program highlights the challenges of keeping learners engaged in self-directed web-based learning.

## Appendix

Multimedia Appendix 1 Survey questions embedded within the Learning Management System.

Multimedia Appendix 2 Four-month implementation survey questions emailed to learners who completed all modules and the post-training survey within the Learning Management System.

## Abbreviations

LMS: Learning Management System
NRS: numeric rating scale
OA: osteoarthritis
RE-AIM: Reach, Effectiveness, Adoption, Implementation, and Maintenance
