# Supplementary material for: An e-Learning Program for Physiotherapists to Manage Knee Osteoarthritis Via Telehealth During the COVID-19 Pandemic: Real-World Evaluation Study Using Registration and Survey Data
Source: JMIR Med Educ. 2021 Dec 1;7(4):e30378. doi: 10.2196/30378 (PMC8686455; doi:10.2196/30378)
Supplement: Multimedia Appendix 1 [file mededu_v7i4e30378_app1.docx]

**APPENDIX 1 –Survey questions embedded within the Learning Management System.**

**^indicates questions asked pre-training only**

***indicates questions asked pre-and post-training**

**†indicates questions asked post-training only**

**^**1. Please indicate your main work role:

- Physiotherapist delivering clinical care to patients
- Other health professional delivering clinical care to patients
- Education of physiotherapy students
- Education of other health professional students
- Physio researcher
- Other health professional researcher
- Physiotherapy student
- Other health professional student
- Other

**^**2. Which of the following health professionals are you?

- Physiotherapist/physical therapist
- Chiropractor
- Rheumatologist
- General practitioner/family physician
- Sport and Exercise Medicine Physician
- Orthopaedic surgeon
- Dietician
- Podiatrist
- Radiologist
- Other

**^**3. Where do you live?

- Africa
- Asia
- Australia
- Europe
- North America
- Pacific Islands (e.g. New Zealand, Fiji, Tonga)
- South America

**^**4. How many patients do you treat, on average, in a month with knee osteoarthritis?

**^**5. How many years of clinical practice experience do you have?

**^**6. How do you usually manage your patients with knee osteoarthritis (select as many as apply)?

- Education
- Exercise
- Physical activity advice
- Weight loss advice
- Manual therapy
- Acupuncture
- Bracing
- Shoe orthotics
- Other (please explain)

**^**7. Do you currently offer video-conferencing consultations to your patients with knee osteoarthritis?

Yes No

**^**8. How much experience do you have with telerehabilitation consultations using video-conferencing?(answered via a 10 point numerical rating scale (NRS))

1 = no experience at all
10 = extremely experienced

*9. How confident are you to undertake telerehabilitation consultations using video-conferencing? (10 point NRS)

1 = not confident at all
10 = extremely confident

*10. How confident are you to undertake telerehabilitation consultations with people with knee osteoarthritis using video-conferencing? (10 point NRS)

1 = not confident at all
10 = extremely confident

*11. How likely are you to use education in a treatment plan for patients with knee osteoarthritis? (10 point NRS)

1 = not at all likely
10 = extremely likely

*12. How likely are you to use strengthening exercise in a treatment plan for patients with knee osteoarthritis? (10 point NRS)

1 = not at all likely
10 = extremely likely

*13. How likely are you to incorporate a tailored general physical activity program in a treatment plan for patients with knee osteoarthritis? (10 point NRS)

1 = not at all likely
10 = extremely likely

^14. How did you find out about the PEAK training program?

- Work colleague
- Friend/family
- Patient
- Twitter
- Facebook
- LinkedIn
- Professional Organisation
- Internet/Website
- Peer-reviewed article
- Magazine/News Outlet
- Physiotherapy Course Instructor
- Other Health Professional Instructor
- Other

**†**15. How long (approximately) did it take you to complete the PEAK program training modules? (hours/minutes)

**†**16. How useful did you find the PEAK Training Program? (10 point NRS)

1 = not useful at all
10 = extremely useful
