# Supplementary material for: An e-Learning Program for Physiotherapists to Manage Knee Osteoarthritis Via Telehealth During the COVID-19 Pandemic: Real-World Evaluation Study Using Registration and Survey Data
Source: JMIR Med Educ. 2021 Dec 1;7(4):e30378. doi: 10.2196/30378 (PMC8686455; doi:10.2196/30378)
Supplement: Multimedia Appendix 2 [file mededu_v7i4e30378_app2.docx]

**APPENDIX 2 –Four-month implementation survey questions emailed to learners who completed all modules and the post-training survey within the Learning Management System**

**Section 1 – About yourself**

**1) What is your email address? (optional)**

**__________________________**

**2) Are you….**
❑ Male
❑ Female
❑ Do not wish to disclose

**3) How old are you?**

❑ Under 30 years

❑ 31-40 years

❑ 41-50 years

❑ 51-60 years

❑ 61-70 years

❑ 71+ years

**4) Please indicate which of the following best describes your main work role:**

1. Physiotherapist delivering clinical care to patients
2. Other health professional delivering clinical care to patients
3. Educator of physiotherapy students
4. Educator of other health professional students
5. Physiotherapist engaged in research
6. Other health professional engaged in research
7. Physiotherapy student
8. Other health professional student

*If selected (a) or (b):*

**Which healthcare setting do you work in? (Please tick all that apply):**

- Private practice
- Acute care hospital
- Rehabilitation hospital
- Community health centre/setting
- Veterans affairs setting
- Other (please explain)

*Plus - If selected (a):*

**What is your main area of clinical practice?**

- Musculoskeletal
- Neurological
- Cardiopulmonary
- Paediatrics
- Gerontology
- Occupational health
- Women’s, men’s & pelvic health
- Aquatic therapy
- Other (please describe)

**Section 2 –Implementation of what you learned with PEAK Training, including your use of downloadable resources.**

As a reminder, the PEAK (Physiotherapy Exercise and physical Activity for Knee osteoarthritis) Training Program was devised by physiotherapists at the University of Melbourne on the basis of research evidence. It guides physiotherapists in how to implement best-practice care to people with knee osteoarthritis, delivered over 5 one-to-one consultations, via video-conferencing (using the Zoom platform) or during face-to-face 'in-person consultations. The PEAK program focuses on patient empowerment through education, prescription of a strengthening exercise program and a physical activity plan, individualised to patient needs, and using minimal exercise equipment (e.g. elastic resistance bands and/or body weight) to enable patients to perform exercise independently at home.

The PEAK program training modules cover evidence-based management of knee osteoarthritis and telehealth delivery via video-conferencing (with Zoom), and provide a semi-structured outline for physiotherapists to deliver education, strengthening exercises, and a physical activity plan across 5 one-to-one consultations. Many of the principles and concepts are applicable to other common musculoskeletal conditions, and to other telehealth video-conferencing platforms.

The Training was comprised of three online modules i) Evidence-Based Knee Osteoarthritis ii) Telehealth Delivery and iii) PEAK Program, as well as a range of downloadable patient booklets (Preparing for your Consultation, Osteoarthritis Information, Exercise Book, Knee Plan and Log Book) and clinician resources (Zoom Troubleshoot Guide, Initiating and using Zoom for video consultations, Accessing the website of exercise videos, pre-consultation survey, Consultation Outline, Readiness Checklist) that were made available for you to use for free at completion of the training program. The PEAK program also provided you with access to a website of PEAK exercise videos.

**1) Did you recommend the PEAK Training Program and/or downloadable resources to anyone else?**

❑ Yes

❑ No

**2) How useful did you find the PEAK Training Program overall?**

1 = Not at all useful

2 = Somewhat useful
3 = Moderately useful
4 = Extremely Useful

**3) How useful did you find the downloadable patient and clinician resources included in the PEAK Training Program?**

1 = Not at all useful

2 = Somewhat useful
3 = Moderately useful
4 = Extremely Useful

**4) How useful did you find the PEAK Exercise Video Library of exercise videos?**

1 = Not at all useful

2 = Somewhat useful
3 = Moderately useful
4 = Extremely Useful

**5) To what extent did the PEAK Training Program change or inform your clinical/research/educational practices?**

1=Not at all
2= to a minor extent
3=to a moderate extent
4=to a large extent

**6) Have you incorporated anything that you learned from the PEAK Training Program into your clinical practice/student clinical placements/educational programs/teaching materials/research activities? Please tick as many as apply:**

(a) I use(d) some/all of the downloadable patient information booklets

(b) I use(d) some/all of the downloadable clinician resources

(c) I use(d) some/all of the downloadable resources as a guide to adapt/create my own personal resources/booklets

(d) I translated some/all of the downloadable resources into another language for use in my clinical practice/teaching/research

(e) I use(d) the PEAK Exercise Video Library of exercise videos

(f) I use(d) the PEAK program to structure/inform my face-to-face in-person consultations/teaching/research with my knee OA patients

(g) I use(d) the PEAK program to structure/inform my telehealth consultations/teaching/research with my knee OA patients

(h) I use(d) the PEAK program to structure/inform my group exercise/education classes/teaching/research with my patients

(i) I use(d) what I learned about video-conferencing to improve my telehealth consultations/teaching/research

(j) Other (open text box to explain)

(k) I have not incorporated anything that I learned from the PEAK Training Program into my clinical practice

If selected (d):

**Which language did you translate the resources into?** (dropdown list)

**7) Prior to completing the PEAK Training Program, were you offering video conferencing consultations to your patients/teaching students about telehealth/researching telehealth?**

❑ Yes

❑ No

If selected ‘yes’:

**How useful was the PEAK Training Program in helping you with your video-conferencing consultations/teaching/research?**

1 = Not at all useful

2 = Somewhat useful
3 = Moderately useful
4 = Extremely Useful

If selected ‘no’:

**Since completing the PEAK Training Program, have you started offering video conferencing consultations to your patients/teaching students about telehealth/researching telehealth?**

❑ Yes

❑ No

If selected ‘yes’:

**How useful was the PEAK Training Program in helping you with your video-conferencing consultations/teaching/research?**

1 = Not at all useful

2 = Somewhat useful
3 = Moderately useful
4 = Extremely Useful
